# Vitamin D and Its Role in Rheumatic Diseases

**DOI:** 10.3390/metabo15040259

**Published:** 2025-04-09

**Authors:** Maritza Vidal, Nancy E. Lane

**Affiliations:** 1 Centro de Diagnóstico de Osteoporosis y Enfermedades Reumáticas (CEDOR), Lima 15036, Peru; 2 Center for Musculoskeletal Health, University of California at Davis School of Medicine, Sacramento, CA 95817, USA

**Keywords:** vitamin D, 25OHD, calcidiol, cholecalciferol, immune system, rheumatic diseases

## Abstract

Vitamin D is a fat-soluble molecule with pleiotropic effects, acting as a steroid hormone on three main organs: the intestine, bone, and kidney. Among its best-known functions at the skeletal level are regulating bone metabolism and mineralization. In 1983, the presence of vitamin D receptors on the surface of immune cells was described, which led to the discovery of new functions on immunological and inflammatory processes. Currently, we know that vitamin D modulates the adaptative immune system by suppressing cells that produce inflammatory cytokines by downregulation, acting as an important regulator of immunity and the inflammatory response. In this article, we will review the synthesis, metabolic pathways, and the role of vitamin D in rheumatic autoimmune diseases.

## 1. Introduction

Vitamin D exerts effects akin to steroid hormones by regulating bone metabolism at a systemic level while interacting with various other hormones. Furthermore, it functions locally as a cytokine, modulating immune responses, as well as influencing cellular differentiation and proliferation, thereby underscoring its significant extra-skeletal actions [1,2]. Vitamin D is primarily known for its role in regulating calcium and phosphate metabolism to maintain bone health; however, in recent years, it has become evident that vitamin D is also essential for proper immune function. The active form of vitamin D, 1,25-dihydroxyvitamin D (calcitriol), interacts with the vitamin D receptor (VDR) found on various immune cells, such as T cells, B cells, and dendritic cells. This interaction modulates both innate and adaptive immune responses [3]. Specifically, vitamin D influences the production of cytokines, the activation of immune cells, and the expression of antimicrobial peptides, which contribute to the body’s defense against infections [2,4]. The immunomodulatory role of vitamin D is particularly significant in the context of autoimmune diseases, including chronic inflammatory rheumatic disorders. Patients with these conditions commonly present with decreased levels of vitamin D, and evidence suggests that this deficiency may contribute to the exacerbation of disease activity and progression [5,6]. In rheumatoid arthritis, vitamin D deficiency has been linked to higher disease activity, whereas in patients with systemic erythematous lupus, a similar relationship has been noted with regard to disease flares and the increased risk of complications [7,8]. Its ability to modulate T cell differentiation and pro-inflammatory cytokine production is of particular interest in understanding how it may help manage conditions driven by immune system dysfunction [9]. Vitamin D plays a crucial role in immune function and has significant implications for the management of rheumatic diseases. The relationship between vitamin D levels and autoimmune disease activity highlights the potential for vitamin D as both a biomarker and a therapeutic target in these disorders. This review will focus on the relationship between vitamin D and disease activity and progression in chronic inflammatory rheumatic conditions.

## 2. Vitamin D Synthesis and Metabolic Pathways

**Figure 1:**
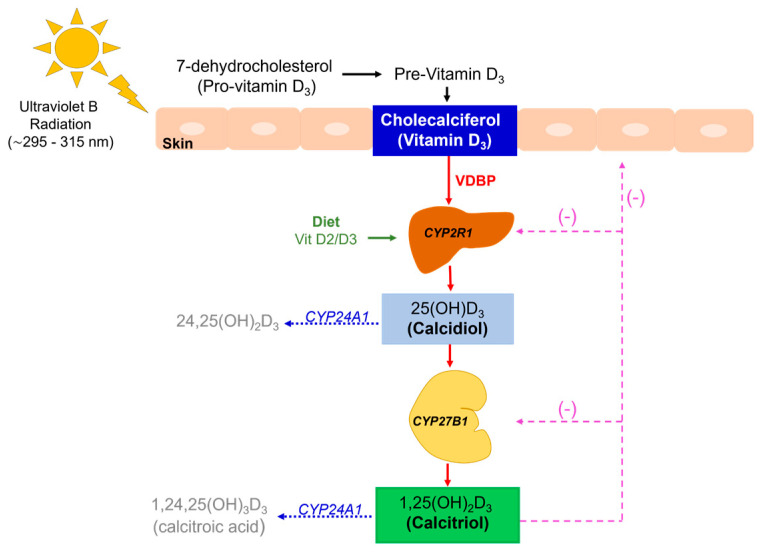
Vitamin D synthesis and activation pathway. VDBP: vitamin D-binding protein; CYP2R1: 25-hydroxylase enzyme; CYP27B1: 1-α-hydroxylase enzyme; CYP24A1: 24-hydroxylase enzyme. After ultraviolet B (UVB) radiation with wavelengths of approximately 295–315 nanometers (nm) in the skin, 7-dehydrocholesterol is converted to pre-vitamin D3 followed by conversion to cholecalciferol by thermal isomerization. Diet can provide vitamin D2 (ergocalciferol) and D3 in minor amounts. Cholecalciferol is transported to the liver bound to VDBP, where CYP2R1 and CYP27A1 enzymes are responsible for its first hydroxylation forming calcidiol, which afterwards is transported to the kidney for a second hydroxylation through CYP27B1 to form calcitriol (1,25(OH)_2_D_3_), the active form of vitamin D. CYP24A1 is responsible for catabolism of vitamin D into 24,25(OH)D_3_ and 1,24,25(OH)_2_D_3_, respectively. Levels of calcitriol are determinants for the self-regulation by this mechanism.

There are two known forms of vitamin D, the first called cholecalciferol from animal origin (which we refer to as vitamin D3), and the second, ergocalciferol, from vegetable sources (vitamin D2). Both forms share the same metabolic pathway. The steroid precursor in animals is 7-dehydrocholesterol (provitamin D3), while in plants and fungi, it is ergosterol. Sun exposure represents the main source of vitamin D in humans, as ultraviolet B sunlight photolyzes 7-dehydrocholesterol (provitamin D3) into pre-vitamin D3, which then undergoes a thermally induced transformation to vitamin D3 (cholecalciferol) [1] (See Figure 1).

Vitamin D-binding protein (VDBP) belongs to the albumin gene family and is mainly produced in the liver, and has a high affinity for vitamin D3 metabolites [10,11]. Two-stage hydroxylation are required to synthesize the active form of vitamin D. After cholecalciferol is formed, VDBP binds and transports it to the liver, and various immune tissues, to undergo a first hydroxylation in position 25 by cytochrome P450 enzymes (CYP2R1, CYP27A1) to produce 25OHD (calcidiol), which represents the major circulating form of vitamin D reflecting the status of this metabolite [1,11] (See Table 1). Calcidiol is transported to the kidney for a second hydroxylation through the 1-α-hydroxylase enzyme (CYP27B1) in the proximal convoluted tubule, generating 1,25(OH)_2_D_3_ (calcitriol), the active form of vitamin D. In clinical practice, measurement of circulating 25OHD is preferred to diagnose vitamin D status since calcitriol has a short half-life of less than 6 h, its hydroxylation in the kidney is regulated by several factors, and its levels can be normal or even increased in patients with vitamin D deficiency due to the stimulus of parathormone (PTH) [1,12]. Calcitriol levels can be self-regulated; for example, in cases where concentrations are high, 24-α-hydroxylase (CYP24A1) enzyme is activated. It catabolizes vitamin D by further hydroxylating calcidiol and calcitriol to 24-hydroxylated products [24,25(OH)D_3_ and 1,24,25(OH)_2_D_3_, respectively], which are then eliminated through the bile and into the feces [1,13,14,15]. When calcitriol levels are low, PTH secretion is stimulated and the vitamin D-catabolic metabolism is inhibited [16].

It is worth noting that extra-renal activation of vitamin D can occur due to the presence of 1-α-hydroxylase in many other tissues such as the skin, lymph nodes, cells of the immune system, parathyroid gland, intestinal epithelium, prostate, pancreas, placenta, adrenal medulla, endometrium, and breast [17,18,19,20]. In these tissues, vitamin D production would perform a paracrine or autocrine action and may be induced by different mechanisms to modulate the cell function [17,18]. For example, in keratinocytes and macrophages, tumor necrosis factor alfa (TNFα) and interferon gamma (IFNɣ) stimulate the 1,25(OH)_2_D_3_ production, and the same occurs in the parathyroid glands, where FGF-23 induces the expression of 1-α -hydroxylase and decreases the secretion of PTH [21].

## 3. Vitamin D Receptor (VDR) and Immune Activation

**Figure 2:**
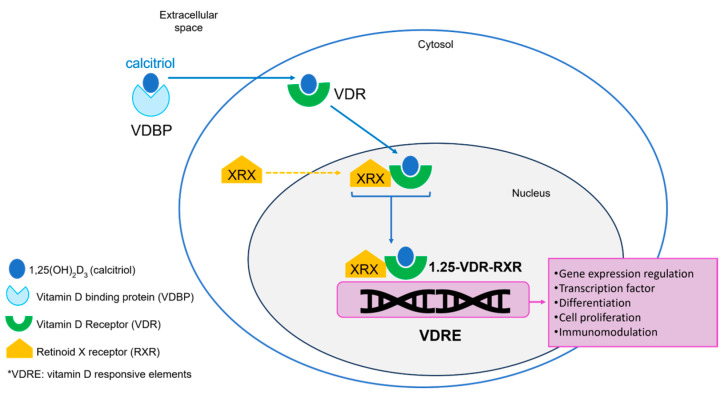
Vitamin D and vitamin D receptor action mechanism (adapted from references [30,31,32]).

Vitamin D exert its functions by binding to the vitamin D receptor (VDR), a member of the superfamily of nuclear receptors and a transcription factor. This union induces changes in the receptor leading to hetero-dimerization with retinoid X receptor (RXR), forming the complex [calcitriol-VDR-RXR] that translocates to the nucleus to bind vitamin D responsive elements (VDREs) in the regulatory regions of vitamin D target genes, acting as a transcription factor [22,23,24,25]. VDR is constitutively expressed by cells of both the innate and adaptive immune system, including macrophages, dendritic cells, B cells, and T cells [26,27]. Dendritic cells and macrophages also express α1-hydroxylase and 25-hydroxylase, suggesting that 25OHD and 1,25(OH)_2_D_3_ production corresponds to an autocrine and paracrine regulation and vitamin D acts as a modulator of the immune system [26,27]. Interestingly, keratinocytes in the skin express VDR, and their production of vitamin D can be regulated in response to calcitriol concentrations [28,29] (See Figure 2).

1,25(OH)_2_D_3_ signaling has a significant impact on immunity by regulating both innate and adaptative immune responses, so understanding the underlying mechanisms will have positive effects in the therapeutic management of many autoimmune conditions [22]. The innate immune system constitutes the first line of defense against microorganisms and is regulated by the production of antimicrobial peptides such as cathelicidins, which participate in the elimination of pathogenic organisms. These substances are produced after toll-like receptors (TLRs’) activation in monocytes, and are regulated by the gene CAMP (cathelicidin antimicrobial peptide), which regulates the innate immune resistance against viruses exerting antibacterial and antifungal activity [3,4,33,34]. These properties make it a crucial regulator of immunity and the inflammatory response. CAMP is also expressed in other cells as lymphocytes, natural killer and dendritic cells [35]. Calcitriol is a pivotal regulator of CAMP acting in many tissues; for example, 1,25(OH)_2_D_3_ and TLR2/1 activation mediates CAMP concentration to act on the elimination of Mycobacterium tuberculosis by monocytes [33,35]. The antibacterial activity of epithelial cells in the lungs is also induced by 1,25(OH)_2_D_3_ and CAMP, which interfere with bacterial cell membranes and generate an immune response. This demonstrates that cathelicidin mediates the immune system at multiple levels [33].

**Figure 3:**
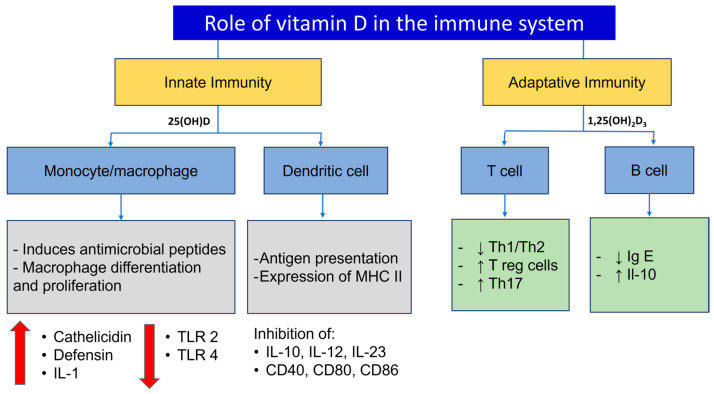
Actions of vitamin D of the immune system. Adapted from References [36,37,38]. IL: Interleukin, TLR: Toll-like receptor, MHC: major histocompatibility complex, Th: T-helper, Ig: Immunoglobulin.

As mentioned above, vitamin D exerts an important role by mediating the innate immune system, regulating inflammatory cells and signals, and acting as an important suppressor of the adaptive immune system, maintaining an immunological balance. Vitamin D modulates the adaptative immune system by suppressing cells that produce inflammatory cytokines by downregulation of the Th1 response and shifting towards a Th2 response; this results in a diminished production of IL-2 and interferon gamma (IFNγ). Calcitriol increases the production of anti-inflammatory interleukins (IL-4, IL-5, IL-10) and decreases the expression of major histocompatibility complex (MHC) class II molecules. Furthermore, calcitriol may moderate the tissue damage caused by inflammation by preventing an exaggerated immune response, promoting tolerance by inducing regulatory T (Treg) cells [9,12,26,35] (See Figure 3).

## 4. Vitamin D in Rheumatic Diseases

**Figure 4:**
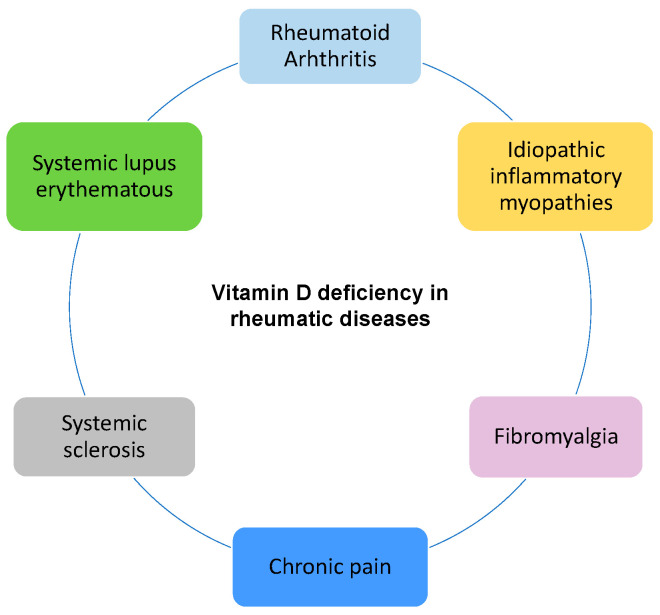
Vitamin D deficiency affects a wide spectrum of rheumatic diseases. Low levels of 25OHD can influence the onset, course, and severity of the disease, as well as predispose to a lower response to treatment and greater impact on the quality of life of patients.

As mentioned, vitamin D exerts an immunomodulatory effect on the innate and adaptive immune system, so it is expected that suboptimal levels would lead to greater disease activity in patients with autoimmune conditions. Vitamin D is reported to suppress B cell proliferation and differentiation into memory and plasma B subtype cells, resulting in the inhibition of immunoglobulin production [6,12,36,39,40]. Several studies have shown that insufficient levels of vitamin D [serum 25OHD] have a positive relationship with the onset, exacerbation, or development of autoimmune diseases; in fact, in cases of vitamin D deficiency, the number and activity of the Treg cells is decreased, predisposing individuals to develop autoimmune diseases [41]. In addition, supplementation with high doses of vitamin D and the normalization of insufficient levels in animal studies has been reported to modify the course of autoimmune diseases, such as rheumatoid arthritis (RA) and systemic lupus erythematosus (SLE) [6,12,42,43,44] (See Figure 4).

### 4.1. Rheumatoid Arthritis

Matrix metalloproteinases (MMPs) are remodeling endopeptidases that regulate components of the extracellular matrix and play a significant role in rheumatic diseases for their capacity for breaking down collagen [45,46]. Matrix metalloproteinases 2 and 9 (MMP-2 and MMP-9), also called gelatinases A and B, are synthesized by chondrocytes and synovial fibroblasts and play a crucial role in joint cartilage damage [47,48] by degrading the extracellular matrix components, the type IV-collagen fibers, and by affecting the chondrocyte function [48]. Thus, their overexpression is related to the development of chronic degenerative diseases such as RA by stimulating the proliferation, migration, and survival of fibroblasts in the joint [45,49]. Recent studies have shown that MMP-2 and MMP-9 are expressed in patients with RA and osteoarthritis, contributing to the progression of structural joint changes; MMP-9 specifically is associated with inflammation and degradation of the cartilage [49,50]. Interestingly, vitamin D deficiency and insufficiency have been found to be associated with higher levels of MMP-2 and MMP-9 [51,52]. Thus, vitamin D supplementation in these groups of patients has been shown to be effective in reducing pain, inflammation, and chondrocyte destruction by lowering MMP concentrations both in vitro and in vivo while also regulating their expression [48,53,54].

Cutolo et al. evaluated the association between vitamin D levels and RA activity in two European groups of women during winter and summer. They found a significantly negative correlation between levels 25OHD and the 28-joint Disease Activity Score (DAS28) among patients from northern Europe in winter and southern Europe in summer, showing that vitamin D among other factors might be implied in the prevalence of RA in these groups [5]. Another study compared 25OHD levels in a group of 44 patients with RA versus a control group, and found that vitamin D levels were significantly lower in patients with the disease than in the control groups (15.26 ± 1.07 ng/mL and 25.8 ± 1.6 ng/mL, respectively) (Student’s *t*-test, *p* < 0.001). Additionally, a significant inverse correlation was found between 25OHD and disease activity levels measured by DAS 28 (correlation coefficient: −0.084) as well as with C-reactive protein (CRP) and erythrocyte sedimentation rate (ESR) values (coefficient being –0.115 and −0.18, respectively), showing that lower 25OHD levels are associated with a higher prevalence and severity of RA [55].

Lin J. et al. analyzed the relationship between 25OHD levels and clinical/laboratory indices of RA disease activity in 24 studies involving 3489 patients. They found an inverse relationship between serum 25OHD levels and RA disease activity indicators, including DAS28 (r = −0.13, 95% CI: −0.16 to −0.09) and CRP (r = −0.12, 95% CI: −0.23 to −0.00). Additionally, the negative relationship between 25OHD and DAS28 was stronger in subgroups of patients in developing countries and in low-latitude areas [56].

A study conducted on 4793 Japanese patients with RA found a mean (SD) serum 25OHD level of 16.9 ng/mL with a prevalence of 11.5% of severe deficiency defined as 25OHD < 10 ng/mL. Among these patients, some characteristics, such as female gender [odds ratio (95% CI): 2.34 (1.88–2.92); *p* < 0.0001]; younger age [odds ratio (95% CI): 0.72 (0.68–0.77); *p* < 0.0001]; high health assessment questionnaire (HAQ) disability score [odds ratio (95% CI): 1.20 (1.07–1.36); *p* = 0.0028]; and nonsteroidal anti-inflammatory drug (NSAID) use [odds ratio (95% CI): 1.27 (1.11–1.46); *p* = 0.0008] were significantly associated with 25OHD < 20 ng/mL (*p* < 0.01). This study showed that patients with lower levels of 25OHD have a higher rate of disease activity and a greater need for NSAID use. These findings were more frequent in females of a younger age [7].

A European multicenter pilot survey supported by the European League Against Rheumatism (EULAR) was performed in 625 patients with RA and 276 age- and sex-matched healthy subjects to investigate the relationship between vitamin D levels and disease activity, quality of life, and disability. The prevalence of 25OHD deficiency was found in 66% of the cases (25OHD < 20 ng/mL) and only 6% had sufficient values (25OHD > 30 ng/mL) without differences among males and females. When comparing 25OHD levels with indicators of disease activity (DAS28), impact of the disease [Rheumatoid Arthritis Impact Diseases score (RAID)], and HAQ, the authors found a negative correlation in RA patients: DAS28-CRP (*p* < 0.001), RAID (*p* = 0.05), and HAQ (*p* = 0.04) [57].

It would be logical to consider that adequate levels of vitamin D are beneficial for patients with autoimmune diseases, and that supplementation in these patients could prevent the recurrence of the disease. Yang J et al. investigated the relationship between vitamin D levels and the recurrence rate in 377 patients with RA. Patients were separated according to their circulating 25OHD levels (normal vs. deficient), and, subsequently, the deficient group was randomly allocated to receive treatment with or without vitamin D supplements. Alfacalcidiol (0.25 µg, twice daily) was administered in the supplemented group and the recurrence of RA was measured by CRP, ESR, and DAS28 scores; patients were followed for a period of 24 months. The authors found that the recurrence rate of RA was the lowest in patients in the normal vitamin D group (16.7%) compared with the vitamin D-deficient patients, and this difference was statistically significant. Among the subgroups of vitamin D-deficient patients, the recurrence was higher in the non-supplemented subgroup (29.5%) versus the supplemented one (19%), although this difference was not statistically significant. The authors concluded that deficient levels of vitamin D are an important risk factor for the increase in disease activity in patients with RA [58].

Buondonno et al. included 39 female patients with early RA and 31 age-matched controls to evaluate differences in T-helper cell sub-types and osteoclast (OC) precursors in peripheral blood in both groups. Subsequently, cholecalciferol (300,000 IU in a single dose) supplementation was administered to RA patients only (along with methotrexate 15 mg/week and methylprednisolone 2 ± 4 mg/day) in a parallel, randomized double blind, placebo-controlled trial that evaluated clinical and laboratory parameters over 3 months. Patients with early RA showed significantly lower levels of 25OHD and an increased cell number of CD4+/IFNγ+, CD4+/IL4+, CD4+/IL17A+, and CD4+IL17A+IFNγ+, and non-classical OCs precursors. Moreover, TNFα, TGFβ1, RANKL, IL-23, and IL-6 levels were increased. Three months after the intervention with cholecalciferol, the control group significantly increased their 25OHD levels (from 16 ± 4 to 28.7 ± 4.3; *p* = 0.01) compared with the placebo group (16 ± 2.1 to 18 ± 3.4; *p* = 0.08). DAS28, CRP, ESR, and the visual analogue scale (VAS) pain parameters were significantly reduced, while global health and HAQ improved. The authors concluded that standard treatment combined with cholecalciferol supplementation was effective in ameliorating the clinical symptoms and global health in patients with early RA [59].

It has been shown that vitamin D supplementation may improve the outcomes of RA. Gopinath et al. supplemented RA patients with 1,25(OH)_2_D_3_ along with disease-modifying anti-rheumatic drugs (DMARDs) and calcium and found a significantly higher pain relief at the end of 3 months compared with the 62 patients receiving DMARDs and calcium alone [60,61]. A cohort of patients with RA was investigated by Malakooti et al. and it was found that higher concentrations of vitamin D (25OHD > 20 ng/mL) were associated with lower subsequent mortality. Patients with higher vitamin D levels before starting methotrexate were found to have a 28% reduced risk of mortality compared with patients with lower levels (HR: 0.72, CI: 0.64–0.80, *p* < 0.001) after adjusting for traditional risk factors [62]. These results support the benefit of combining DMARDs and vitamin D in patients with RA, particularly in those with insufficient vitamin D levels before starting treatment.

### 4.2. Systemic Lupus Erythematosus

Kamen et al. compared circulating levels of 25OHD in 123 recently diagnosed SLE patients and 240 matched controls and found that 67% of the SLE patients were vitamin D deficient (25OHD < 30 ng/mL), with levels significantly lower among African Americans (15.9 ± 9.4 ng/mL) compared to Caucasians (31.3 ± 14.9 ng/mL; *p* < 0.01). A lower concentration of 25OHD was associated with discoid rash among Caucasian patients (*p* < 0.01). Additionally, 18% of the SLE patients had very low vitamin D levels (25OHD < 10 ng/mL) with renal disease as the strongest predictor (OR 13.3, 95% CI: 2.3–76.7, *p* < 0.01) followed by photosensitivity (OR 12.9, 95% CI: 2.2–75.5, *p* < 0.01), concluding that deficient levels of 25OHD could represent a risk factor for developing lupus [63].

A systematic review and meta-analysis by Guan included 19 articles comparing vitamin D levels in SLE patients and healthy controls. Results showed lower levels of 25OHD in the patient group (pooled SMD = −1.63, 95% CI: −2.51 to −0.76), with significant heterogeneity among these studies (I2 = 98.9%, *p* < 0.001). After a subgroup analysis, it was found that these lower values were influenced by Arab and European ethnicity; activity of the disease was assessed with the Systemic Lupus Erythematosus Disease Activity Index (SLEDAI) ≥ 10 instrument (pooled correlation coefficient = −0.50, 95% CI: −0.8278 to −0.1689) [8]. Another research group also reported a relationship of lower levels of 25OHD in high-activity SLE patients evaluated by SLEDAI (R= −0.65, *p* < 0.001); they also found a higher prevalence of vitamin D deficiency among SLE patients compared with healthy controls [64].

Zou et al. investigated the relationship between vitamin D levels and SLE disease activity (SLEDAI) in 158 patients and 50 healthy controls. SLE patients were divided into two subgroups according their SLEDAI scores (active vs. non active groups). Mean levels of 25OHD were significantly lower in the SLE patients vs. the control group (10.4 vs. 25.5 ng/mL; *p* < 0.01, respectively). Among SLE patients, those with active disease had lower 25OHD values compared with those in remission (6.2 vs. 12.3 ng/mL; *p* < 0.01). Lower values were also found in patients with lupus nephritis compared to those without renal involvement (6.7 vs. 13.3 ng/mL; *p* < 0.01, respectively). Interestingly, a negative correlation was found between serum 25OHD and SLEDAI (r = −0.35, *p* < 0.01) and the 24-h urinary protein excretion (r = −0.39, *p* < 0.01), showing that 25OHD levels in SLE patients are important in the course of the disease and normalizing these levels could help prevent the progression of disease activity in these patients [65].

Attar et al. evaluated 95 patients with SLE and found an overall vitamin D deficiency in 87% of them. SLE activity was evaluated by SLE Disease Activity Index 2000 (SLEDAI-2K) and was considered as active disease if the score was >4, or inactive if <4. Patients with active disease were found to have significantly lower mean 25OHD values than those with inactive SLE (8.9 vs. 10 ng/mL; *p* = 0.04), although no statistical correlation was found. A significant negative correlation was found between 25OHD levels and anti-double stranded DNA (dsDNA) antibodies (r = −0.38; *p* < 0.001); additionally, 25OHD levels and C4 were found to have a positive correlation (r = 0.25; *p* = 0.25) [66]. Shevchuk et al. evaluated the relationship between vitamin D levels and disease activity in 101 SLE patients and 29 healthy controls. The mean 25OHD level was 18.98 ± 0.88 ng/mL with lower levels among women compared to men (*p* < 0.05). Lower levels of vitamin D were associated with increased inflammatory activity, shown by an inverse correlation between 25OHD and CRP (r = −0.39), IL-6 (r = −0.37), and ESR (r = −0.15); additionally, an inverse correlation with a cumulative dose of glucocorticoids was found (r = −0.26) [67].

### 4.3. Systemic Sclerosis

Systemic sclerosis (SSc) is a complex autoimmune disease characterized by progressive widespread fibrosis and vascular abnormalities [68,69]. This specific group of patients present thickening, fibrosis, and hyperpigmentation of the skin, which along with their reduced sun exposure, constitute a risk for vitamin D deficiency due to the lack of cutaneous synthesis [70,71]. Esophageal dysmotility and gastroparesis make it difficult for patients to take vitamin D supplements; similarly, bacterial overgrowth in the small intestine causes vitamin D malabsorption, contributing to these patients’ risk of developing hypovitaminosis [72].

Groseanu et al. evaluated 51 patients with SSc according the European League Against Rheumatism 2013 and analyzed their disease involvement (visceral compromise, immunological profile, activity and severity scores, and quality of life). Vitamin D insufficiency and deficiency was found in 66.66% and 23.52%, respectively, with only 9.8% of the patients with optimal levels. The mean 25OHD level was 17.06 ± 9.13 ng/dL. A negative correlation was found between 25OHD and pulmonary hypertension (*p* = 0.053, r = −0.29), muscle weakness (*p* = 0.015, r = −0.377), digital contractures (*p* = 0.036, r = −0.298), and diastolic dysfunction (*p* = 0.033, r = −0.318), and a positive diffusing capacity of the lung for carbon monoxide (*p* = 0.019, r = 0.353). This led to the conclusion that lower 25OHD levels are related to pulmonary and cardiac involvement in patients with SSC [71]. In addition, a systematic review of 40 publications reported an increased prevalence of hypovitaminosis D in patients with SSC, and a possible relationship between lower levels and serologic markers of the disease [68].

A metanalysis by Sun et al. included 554 SSc patients and 321 healthy subjects, and showed that patients had lower vitamin D levels than controls (SMD =−8.72 ng/mL; 95% CI: −10.11 to −7.32); this was especially evident in diffused-type SSc patients compared with those with the limited type (SMD =−4.71 ng/mL; 95% CI: −8.98 to −0.44). There was no association between vitamin D levels and the severity of the disease: Rodnan score (SMD =−2.29 ng/mL, 95% CI: −8.49 to 3.91, *p* = 0.47), pulmonary involvement (RR =1.01, 95% CI: 0.36–2.86, *p* = 0.99), systolic pulmonary pressure (SMD =−1.68 ng/mL, 95% CI: −10.79 to 7.43, *p* = 0.72), or gastrointestinal ulcer (RR = 1.01, 95% CI: 0.53–1.93, *p* = 0.98) [73].

Caramaschi et al. found a mean 25OHD level of 15.8 ± 9.1 ng/mL in 65 SSc patients with a prevalence of 29.2% patients with levels below 10 ng/mL and 66.1% between 10 and 30 ng/mL, respectively. When analyzing other variables, the investigators found that levels < 10 ng/mL showed longer disease duration (13.1 ± 6.8 versus 9.4± 5.5 years, *p* = 0.026), lower diffusing lung capacity for carbon monoxide (63.7 ± 12.4 versus 76.4 ± 20.2, *p* = 0.014), higher estimated pulmonary artery pressure (28.9 ± 9.9 versus 22.8 ± 10.4, *p* = 0.037), and increased values of ESR (40 ± 25 versus 23 ± 13 mm/h, *p* = 0.001) and of CRP (7 ± 7 and 4 ± 2 mg/L, *p* = 0.004) compared with patients with higher vitamin D levels [74].

Dhaouadi et al. also found lower levels of vitamin D among patients with SSc compared with healthy controls with raw mean differences 95% CI = −11.68 [−15.43 to −7.92] ng/mL, *p* < 1 × 10^−10^. When analyzing the occurrence of interstitial lung disease in patients with low vitamin D levels, a significant association was found (raw mean differences 95% CI = −3.61 [−6.93 to −0.3], *p* = 0.003); also, there was an association between low 25OHD and increased systolic pulmonary arterial pressure (raw mean differences 95% CI = 4.17 [1.44–6.89], *p* = 0.003). Finally, there was a negative correlation between vitamin D levels and the modified Rodnan skin score, *r* (95% CI = −0.26 [−0.44 to −0.08], *p* = 0.004) [75]. Despite the high prevalence of insufficient vitamin D levels in patients with SSc, further clinical studies are required to evaluate whether correction of calcidiol levels may have an effect on the course of the disease, and its clinical impact remains uncertain [76].

### 4.4. Idiopathic Inflammatory Myopathies

A recent meta-analysis evaluated the correlation between vitamin D and idiopathic inflammatory myopathy (IIM) in five studies involving 286 patients and 480 healthy controls and found significantly lower levels of serum 25OHD in IIM patients compared to controls [mean differences (MDs) = −13.10 ng/mL; 95% CI: −16.51 to −9.68; *p* < 0.00001] using a random-effects model. Two other studies that included 185 IIM patients revealed that levels of creatine kinase were higher in subjects with low vitamin D levels, compared with normal levels (MD = 85.20 IU/L; 95% CI: 72.67–97.73; *p* < 0.00001), showing a positive correlation between vitamin D deficiency and increased risk of IIM (RR = 3.24, 95% CI: 1.81–5.79; *p* < 0.0001) [77].

Vitamin D deficiency may be a risk factor for IIM; Azali et al. evaluated circulating levels of 25OHD in 149 IIM patients and 290 healthy controls, and found lower levels in the IIM group compared with the healthy one (median 15.6 vs. 27.2; *p* = 0.0001). The IIM group was subclassified in polymyositis (PM), dermatomyositis (DM), inclusion body myositis (IBM), and juvenile DM (JDM), but no significant difference in vitamin D levels was found among these subgroups. However, when classifying them according to vitamin D status, it was found that vitamin D deficiency (25OHD < 20 ng/mL) was more frequent in the IIM group [PM (68%), DM (65%) and IBM (53%)] compared to healthy controls (21%). The odds of IIM patients with vitamin D deficiency compared to the healthy group were 17.7 (95% CI: 8.1 to 38.6), and for vitamin D insufficiency (25OHD: 20–30 ng/mL) versus healthy patients 2.4 (95% CI: 1.2 to 4.7) [78].

Observational studies have demonstrated the impact of inadequate vitamin D circulating levels in inflammatory and autoimmune rheumatic diseases. Vitamin D not only acts as an immunomodulatory molecule to reduce inflammation but also has a role in enhancing muscle regeneration and mitochondrial function by stimulating satellite cells’ production of myogenic components and inhibiting the myostatin, a protein that negatively regulates muscle mass. These actions support the role of vitamin D in muscle recovery [79,80,81]. Patients with IIM should achieve sufficient levels of vitamin D to ensure proper immune function and reduced disease activity as well as to improve muscle strength, pain, and fatigue [81,82].

### 4.5. Fibromyalgia and Chronic Pain

As we have previously reviewed, vitamin D deficiency is highly prevalent among patients with various autoimmune conditions, suggesting a possible association between insufficient levels and the clinical course. There are other common conditions in clinical practice, particularly in the field of rheumatic diseases, that are also associated with decreased levels of vitamin D. Muscle weakness and generalized bone pain are common manifestations of osteomalacia and myopathy secondary to severe vitamin D deficiency. In many cases, these symptoms may be confused with or attributed to pain-related conditions, such as fibromyalgia. It is important to consider this, especially in patients diagnosed with fibromyalgia, where both pharmacological and non-pharmacological interventions have failed [83].

Recently, Vidal et al. investigated the vitamin D status in 157 Peruvian women ≥50 years of age who were treated at a rheumatology service for the first time, and they found that 58.6% of patients had values of 25OHD < 20 ng/mL, with only 4.5% of patients with sufficient values ≥ 30 ng/mL. 25OHD values were lower in obese patients than in non-obese patients (16.94 vs. 19.42 ng/mL; *p* < 0.05). Among these patients, the most frequent diagnosis was fibromyalgia in 50% and osteoarthritis in 30.57%. Interestingly, no correlation was found between age and 25OHD levels (r = 0.099; *p* = 0.214). This study showed that vitamin D insufficiency is common in rheumatic patients, and correcting it might be beneficial for the management and course of their diseases [84].

Von Känel et al. examined the association between low serum levels of 25OHD and mechanical pain sensitivity in 174 patients with chronic pain. In total, 53% were women with a mean age of 48 years. Pain intensity was rated on a numerical analogue scale (0–10) after a standardized pain provocation test, and a widespread pain index with a symptom severity score from the American College of Rheumatology 2010 preliminary diagnostic criteria for fibromyalgia were also assessed. Circulating 25OHD < 20 ng/mL levels were found in 71% of patients with chronic pain, while 21% showed levels < 30 ng/mL. After adjustment for known and potential covariates, the authors found a mean increase in pain intensity of 0.61 ± 0.25 (mean ± standard error) for each 10 ng/mL decreased in 25OHD. Greater symptom severity (*r* = −0.21, *p* = 0.008) was associated with lower levels of 25OHD, although this was not found in the widespread pain index (*p* = 0.83) and fibromyalgia (*p* = 0.51). The authors concluded that vitamin D might have a role in patients with chronic pain, augmenting the processing of pain upon mechanical stimulation [85].

Gendelman et al. assessed the supplementation of 4000 IU of cholecalciferol or a placebo in 74 patients with musculoskeletal pain for 3 months. Patients were older than 18 years and had pain lasting more than 6 months. In total, 73% of the patients had 25OHD < 30 ng/mL (28 patients in the intervention group and 24 in the placebo arm). Pain parameters were scored prior to intervention, and at week 6 and week 12. Visual analogue scale (VAS) scores of pain perception were recorded following 6 and 12 weeks. The supplemented group showed a statistically significantly larger reduction in the VAS pain scores throughout the study. These patients also showed a lower use of analgesic therapy compared with those with a placebo. Interestingly, the authors measured circulating levels of leukotriene B4 (LTB4), interleukin 6 (IL-6), tumor necrosis factor alpha (TNFα), and prostaglandin E2 (PGE2), and found that the vitamin D-supplemented group’s TNFα levels decreased by 54.3% (mean value at baseline of 10.5 ± 37.4 pg/mL to 4.8 ± 6.4 ng/mL after 6 weeks, *p* < 0.026), while these increased by 16.1% (mean value at baseline of 3.1 ± 1.9 pg/mL to 3.6 ± 2.3 ng/mL after 6 weeks) in the placebo group. Prostaglandin E2 (PGE2) levels also decreased by 39.2% in the vitamin D group (mean value at baseline of 631.4 ± 923.2 pg/mL to 384.2 ± 624 pg/mL after 6 weeks, *p* < 0.008) and increased by 16.1% in the non-supplemented group (mean value at baseline 450.5 ± 532.7 pg/mL to 506.6 ± 656 pg/mL after 6 weeks). LTB4 levels decreased in both groups by 24% (*p* < 0.05). This study showed that supplementing patients with musculoskeletal pain might be beneficial not only in the perception of pain but in the circulating levels of inflammatory cytokines that activate the nociceptive pain pathway [86].

In recent years, the impact of vitamin D on the development and perpetuation of chronic pain has been demonstrated, especially in patients with inflammatory rheumatic diseases, in whom decreased levels of 25OHD are associated with greater disease activity, greater pain severity, and a weakened immune system. Vitamin D also influences pain perception and disease outcome. Vitamin D has an important role in the inhibition or potentiation of neurotransmitters such as substance P, acetylcholine, glutamate, adenosine, GABA, and monoamine, and its implication for sleep disorders, neuropsychiatric conditions, and chronic pain has been described [87]. Further studies are needed to establish the importance of correcting vitamin D deficiency and the benefits that might come from the supplementation of cholecalciferol in this group of patients [86].

## 5. Discussion and Final Remarks

The effects of vitamin D are widely known, especially for its importance in calcium homeostasis, exerting beneficial effects on the musculoskeletal system, and preventing fractures. Vitamin D also has a key role in the regulation of the immune system, exerting a dual effect by boosting innate immunity and suppressing the adaptive inflammatory response [88]. In recent years, an association has been identified between insufficient vitamin D levels and the development of autoimmune diseases, as well as the persistence of inflammation and activity in rheumatic diseases [89]. Vitamin D acts as a regulator of cell proliferation and differentiation in the immune system, so it is important to maintain adequate levels to ensure proper immunomodulation [41]. Vitamin D deficiency is a global problem that must be addressed and corrected in all patients, especially in those with rheumatic inflammatory diseases. As mentioned earlier in this article, inadequate levels of vitamin D have been associated with higher rates of inflammatory disease activity, worse outcomes, and increased systemic inflammation markers [5,55,56,57,58,59,60,61,62,63,64,65,66,67,68,69,70,71,72,73,74,75,76,77,78,79,80,81,82,83,84,85,86]. It is reasonable to consider that vitamin D supplementation and correction of insufficient levels would benefit the outcomes of all patients with rheumatic diseases. Levels of 25OHD above 30 ng/mL are desirable in the general population to maximize the health benefits of vitamin D at the musculoskeletal level [90]. Sufficient levels of 25OHD > 30 ng/mL should be considered in those patients in whom it is desired to strengthen the immune system, especially those with inflammatory rheumatic diseases [61].

## Figures and Tables

**Table 1:** Practical reference definitions of enzymes in the vitamin D metabolic pathway.

Cholecalciferol (vitamin D3)	Fat-soluble molecule produced in the skin after UV light exposure. It is a non-hydroxylated form of vitamin D3
25-hydroxylase enzyme (CYP2R1)	Hepatic enzyme that hydroxylates cholecalciferol into calcidiol
25OHD (calcidiol, calcifediol) 25OHD3 if animal origin 25OHD2 if vegetable origin	Hepatic molecule obtained after the first hydroxylation of cholecalciferol in the liver
1-α-hydroxylase enzyme (CYP27B1)	Renal enzyme that hydroxylates calcidiol into calcitriol
1,25(OH)_2_D (calcitriol) 1,25(OH)_2_D_3_ if animal origin 1,25(OH)_2_D_2_ if vegetable origin	Active form of vitamin D produced after renal hydroxylation
25(OH)D-24-α-hydroxylase 24-hydroxylase 1,25(OH)_2_-D-24-hydroxylase (CYP24A1)	Catabolic enzyme that degrades metabolites of vitamin D into soluble calcitroic acid for rapid renal excretion
1α-hydroxy-23-carboxy-24,25,26,27- tetranorvitamin D_3_ (calcitroic acid)	Inactive product result of the catabolism of calcitriol; it is soluble in water and excreted in bile

## Data Availability

The data presented in this study are available in the text.
